# Notes and News

**Published:** 1892-07-23

**Authors:** 


### Notes and News.

Collected and Communicated.

A new eye infirmary was opened at Durham last week. The building is two storeys high, and provides accommodation for sixteen in-patients, and consists of four wards, two male and two female. Each ward has separate lavatory, &c. There is a good operating room. An increase of beds is possible by utilizing the vacant attics for servants' quarters. Mrs. Backhouse, whose family have been the chief supporters of the movement, declared the buildings open.

An American paper draws attention to life insurance for women. It states that two reasons for the small number of female insurances exist—one that women are not usually the bread-winners of the family, and, secondly, that the companies themselves object to female policy-holders on the ground that women are disposed to die of casualties and diseases from which men are exempt. Passing over the pros and cons. of these arguments, we come to the somewhat startling statement that the mortality of women whose lives are insured in favour of their husbands is larger than the ordinary mortality tables warrant. The writer of the article concludes with the advice to women not to insure in favour of their husbands, and to companies to refuse to issue such a policy.

Miss Weston gives a gratifying account of the growth of her schemes in behalf of sailors in Devonport and Portsmouth. Well-conducted "sailors' rests" are amongst the most useful institutions of the day, a fact well known to those who are acquainted with the ways of the sea-faring classes. Jack is glad to be protected from himself and those who have designs upon him when he comes ashore with well-filled pockets. The "rests" are rapidly growing in popularity. During the year as many as 105,000 sailors now seek shelter in the two "rests" at Devonport and Portsmouth. The accommodation, however, is not sufficient for the demands upon it, and Miss Weston has purchased a house adjoining her present premises for the sum of £1,000. She appeals for assistance to defray the cost of this much-needed purchase.

Last Saturday the annual Hospital Saturday Street Collection took place and amounted to £1,902 in the Central District, an improvement on last year. Further returns are not yet to hand. The services of 2,500 ladies were brought into requisition. Last year the collection amounted to £5,000.

The sixtieth annual meeting of the British Medical Association is to be held at Nottingham, commencing on Tuesday, July 26th. The scientific proceedings are to be divided into ten sections. Numerous entertainments and excursions in connection with the meeting are announced. An exhibition is to be arranged in University College, Nottingham, the exhibits including food, drugs, surgical appliances and dressings, models, drawings, and microscopical preparations.

The annual meeting of the Metropolitan Drinking Fountains, &c., Association was held at the Westminster Town Hall on Wednesday, 13th inst., the Duke of Westminster presiding. His Grace made an urgent appeal for a more generous support of the Society, whose valuable work is known to all. The ordinary income of the Society is quite inadequate, being only £2,476 last year. There are now 690 fountains and 744 troughs under the supervision of the Society, and the cost of maintaining these is over £3,000 per annum—the cost of water alone being nearly £1,600 per annum. Applications for new fountains and troughs had to be refused during the year from want of funds. We heartily commend the Duke's appeal on behalf of this deserving Society.

An interesting ceremony took place at Streatham on the 16th inst., on the occasion of the laying of the foundation-stone of the new British Home for Incurables at Streatham, by the Princess Christian. The scene was a very pretty one, as the event was further celebrated by a floral display and garden party on the site of the new Home. Many of the flowers were contributed by Royal donors, a quantity of roses being sent by the Queen from Frogmore. Her Majesty further sent a contribution of £25 towards the new buildings, and £1,000 was also received from "Two Sisters" to found a bed in the institution. The cost of the new Home is estimated at £22,000, of which £14,000 has been taken from the reserve fund, leaving £8,000 to be collected from public subscriptions.

The recent criticism made by Sir J. Crichton Browne on the female brain has provoked universal comment, but very little approbation. He has brought down upon the school whose curriculum resulted in so colossal an amount of headache in its scholars, a series of condemning remarks. In one paper it is very naturally suggested that the drainage must be defective. An Americen journal remarks that the bad results must be due to "method," and suggests that if high schools in England, as Dr. Browne thinks, "make the female leg thin, the bust flat, and the eye myopic," they must, indeed, need reform, no such unfavourable results attending the same system of education in America. We almost wonder the high schools have not given a statistical refutation of the charges made against one of their number, chosen to represent their method.

